# Multicentric survey on dose reduction/interruption of cancer drug therapy in 12.472 patients: indicators of suspected adverse reactions

**DOI:** 10.18632/oncotarget.8942

**Published:** 2016-04-22

**Authors:** Andrea Casadei Gardini, Elena Tenti, Carla Masini, Oriana Nanni, Emanuela Scarpi, Martina Valgiusti, Silvia Restuccia, Maria Laura Gallani, Simonetta Palazzini, Erica Bianchini, Silvia Menozzi, Antonio Maugeri, Dino Amadori, Martina Minguzzi, Giovanni Luca Frassineti

**Affiliations:** ^1^ Department of Medical Oncology, Istituto Scientifico Romagnolo per lo Studio e Cura dei Tumori IRST IRCCS, Meldola, Italy; ^2^ Oncology Pharmacy Laboratory, IRST IRCCS, Meldola, Italy; ^3^ Unit of Biostatistics and Clinical Trials, IRST IRCCS, Meldola, Italy; ^4^ Department of Clinical Pharmacy, University Hospital S. Orsola-Malpighi, Bologna, Italy; ^5^ Department of Clinical Pharmacy, University of Parma, Parma, Italy; ^6^ Department of Clinical Pharmacy, Azienda Ospedaliera della Romagna (AUSL), Rimini, Italy; ^7^ Department of Clinical Pharmacy, University Hospital of Ferrara, Ferrara, Italy; ^8^ Department of Clinical Pharmacy, Hospital S. Maria Nuova, Reggio Emilia, Italy

**Keywords:** sorafenib, capecitabine, pharmacovigilance, docetaxel, oxaliplatin

## Abstract

Antiblastic drugs have a high number of potential side-effects. Paradoxically, according to the National Network of Pharmacovigilance, the number of reported adverse reactions to these agents is proportionally lower than that registered for non antiblastic drugs. Critical phenomena such as treatment interruptions and significant dose reductions within the first two months of use may be indicators of adverse drug reactions. The aim of the present study was to increase our knowledge of pharmacovigilance to facilitate the actions taken to improve the risk-benefit profile of cancer drugs and, consequently, their safety. This retrospective observational survey was carried out on prescriptions from 1st January 2012 to 31st December 2012. Dose reductions of more than 10% during the first 90 days of therapy were considered as a surrogate indicator of an adverse reaction. Dose interruptions during the first 60 days of therapy were taken into consideration. Of the12,472 patients 1,248 underwent a dose reduction. The drugs that most often required a dose reduction were paclitaxel and oxaliplatin (17.4% and 17.3%, respectively), docetaxel (14.8%), carboplatin (15%), fluorouracil (10.7%) and, among oral medications, capecitabine (6.9%). Of the 1896 patients treated with the same drugs, 9.7% interrupted treatment. Patients required a lower dose reduction than that reported by other authors. Around 15% of cases underwent a 30% dose reduction within three months of starting therapy, indicating a possible adverse reaction. Constant monitoring of dose prescription and continuous training of medical and nursing staff are clearly needed to increase awareness of the importance of reporting adverse events.

## INTRODUCTION

Pharmacovigilance has been defined by the World Health Organization (WHO) as “the science and activities relating to the detection, assessment, understanding and prevention of adverse effects or any other possible drug-related problems” [1]. The correct Adverse drug reaction definition (ADRs) is : reaction noxious and unintended, to medicinal products used at doses normally used in man for the prophylaxis, diagnosis or treatment, correction or modification of physiological functions. Adverse events are unwanted and usually harmful outcomes. The event may or may not be related to the treatment, and is not the same as a side effect or an adverse reaction because it is not always clear whether the drug has caused the event.

In a meta-analysis conducted from 1966 to 1996 on a sample of hospitalized patients, Lazarou and colleagues reported that the overall incidence of serious ADRs was 6.7% (95% confidence interval [CI] 5.2%-8.2%) and that the incidence of fatal ADRs was 0.32% (95% CI 0.23%-0.41%) [2]. They estimated that, in 1994, 2,216,000 hospitalized patients had serious ADRs and 106,000 had fatal ADRs, making these reactions between the fourth and sixth leading causes of death.

Antineoplastic agents have a potentially high number of side-effects. The overall safety profile of cancer drugs is not known, yet this is an element that allows us to monitor the patient's quality of life. For this reason, it is of vital importance to report adverse events, even if these are not serious and have already been documented. A good internal reporting system ensures that all parties involved responsible are aware of major hazards. Reporting ADRs is also essential to monitor the progress made in error prevention [3].

In 2012, around 70,150 expedited adverse reaction reports were received and processed each month and subsequently made available for signal detection and data analysis by the European Medicines Agency (EMA) and the medicine regulatory authorities of Member States. Of these, 15% were reports on cancer drugs [4]. Consequently, an Italian study on ADR reporting was performed by the National Network of Pharmacovigilance [5] and data on cancer-related ADRs reported in 2011 were extrapolated. 21,473 ADRs equivalent to a signaling rate of 356 reportsper million inhabitants were received by the Italian National Network of Pharmacovigilance, and a total of 300 reports per one million people in the population was reached and exceeded, 300 being the figure defined by WHO as the gold standard for an efficient pharmacovigilance system.

European pharmacovigilance legislation also considers a lack of treatment efficacy and medication errors as adverse reactions. Such events are not intrinsic to the patient or the drug, but are related to the management of the correlated processes, which can influence the risk-benefit profile. It is clear that such phenomena in a cancer setting must be closely monitored. It is essential to keep track of critical events such as treatment interruptions and significant dose reductions that occur within the first two months of treatment.

The aim of the present study were also to identify and report suspected adverse durg reactions and increase communication between pharmacist and oncologist for proper overall management of the drug, since the pharmacovigilance system.

## RESULTS

Of the12,472 patients who received one of the study drugs from 1^st^ January 2012 to 31^st^ March 2013, 11,596 were treated intravenously and 876 orally. 1197 (10.3%) patients in the former group underwent a dose reduction during the first 3months of treatment compared to 51 (5.8%) the latter group. Table 1 shows the percentage of patients who required a dose reduction with respect to the entire case series.

**Table 1:** Percentage of dose reductions

Intravenous drugs Active	No. of patients undergoing treatment	No. of patients with dose reduction	%	Rate of reduction 40%-50%≤	10%-20%≤	20%-30%≤	30%-40%≤
Paclitaxel	1208	210	17.4	17	68	96	29
Oxaliplatin	1297	224	17.3	10	95	105	14
Carboplatin	1690	255	15.0	8	160	69	18
Docetaxel	995	147	14.8	3	77	49	18
Fluorouracil	2351	252	10.7	13	104	121	14
Ifosfamide	231	11	4.8	0	5	4	2
Anthracycline	2148	75	3.5	7	51	9	8
Pemetrexed	392	10	2.6	0	10	0	0
Rituximab	1284	13	1.0	0	12	0	1
Total	11596	1197	10.3	58	582	453	104
**Oral drugs Active**	**No. of patients undergoing treatment**	**No. of patients with dose reduction**	**%**	**Rate of reduction 40%-50%≤**	**10%-20%≤**	**20%-30%≤**	**30%-40%≤**
Capecitabine	662	46	6.9	3	23	17	4
Erlotinib	108	3	2.8	0	0	0	3
Sorafenib	47	2	4.3	1	1	0	0
Lenalidomide	59	0	0.0	0	0	0	0
Total	876	51	5.80	4	24	17	7

The most frequent dose reductions in intravenous drugs were made in paclitaxeland oxaliplatin (17.4%and 17.3% of patients, respectively), followed by docetaxel (14.8 %), carboplatin (15%) and fluorouracil (10.7%). Among orally administered drugs, capecitabine showed the highest percentage of dose reduction (6.9%). No dose reductions were required in the 59 patients treated with lenalidomide.

Of the1896 patients who received one of the study drugs from 1^st^ January 2012 to 28^st^ February 2012,1575 were treated intravenously and 321 orally. Treatment interruption due to adverse drug reactions (ADRs) occurred in133 (8.4%) patients of the intravenous group and 51 (15.9%) of the oral group. The highest number of ADRs leading to treatment discontinuation were registered for ifosfamide (12 ADRs in 35 patients) followed by doxorubicin (23ADRs in 164 patients), carboplatin (32 ADRs in 300 patients) and paclitacxel (14ADRs in 147 patients) (Table 2).

**Table 2:** Drugs with highest percentage of clinical reaction leading to discontinuation

Intravenous drugs	No. of patients undergoing treatment	No. of patients with clinical events	%
Ifosfamide	35	12	34.3
Doxorubicin	164	23	14.0
Carboplatin	300	32	10.7
Paclitaxel	147	14	9.5
Epirubicin	22	2	9.0
Docetaxel	133	12	9.0
Oxaliplatin	139	9	6.8
Fluorouracil	302	19	6.3
Pemetrexed	41	2	4.9
Rituximab	292	8	2.7
**Total**	**1575**	**133**	**8.4**
**Oral drugs**			
Lenalidomide	28	7	25.0
Capecitabine	240	41	17.1
Sorafenib	27	3	11.1
Erlotinib	26	0	0.0
**Total**	**321**	**51**	**15.9**
**Total intravenous + oral drugs**	**1896**	**184**	**9.0**

8.7% of patients discontinued treatment during adjuvant therapy, 23.7% of whom during first-line therapy, 13.5% during second-line therapy, 20% during third-line therapy, 11.1% during fourth-line therapy, and 33.3% during fifth-line therapy. Intravenous medication was discontinued in 8.4% of cases (133/1575), while oral medications were discontinued in 15.9% of cases (51/321).

In dose reduction analysis the percentage of patients treated by intravenous drug was 92.7% and 7,3% with oral drugs. In discontinuation analysis the percentage of patients treated by intravenous drug was 83% and 17% with oral drugs.

A total of 242 ADRs were reported by the 7 centers in the National Network of Pharmacovigilance (RNF), representing an increase of 52% compared with the previous year. The main drugs involved were paclitaxel, oxaliplatin, fluorouracil, carboplatin, and bevacizumab. 84% of reported events were considered non-serious and 16% serious.

## DISCUSSION

The aim of pharmacovigilance is to assess the post-marketing safety of drugs. In oncology, adverse drug reactions are still underreported. Ten percent of patients in the underwent dose reduction and 9.7% of patients interrupted treatment. The reduction of intravenously drug (10.3%) was almost twofold that oral target (5.8%). This is because, according to our data, the targeted therapy drugs are reduced to a smaller percentage compared to the data obtained from the studies. For example, in a study of 84 non-small-cell lung cancer patients treated with erlotinib, the drug was reduced in 21% of cases experiencing grade 3 or 4 adverse events [6]. We treated 108 patients with erlotinib but only experienced three dose reductions amounting to 2.8% of receiving this drug. Sorafenib, another oral drug, was reduced in 4.3% of our patients, whereas the SHARP trial [7–8] reported that 9.4% of patients experienced toxicity ≥ grade 3 protocol amendment.

In our study, 13.5% of patients who reduced the dose of chemotherapy within three months of starting treatment underwent a reduction of more than 30%. Some drugs, *e.g.* paclitaxel, were reduced in 21.9% of patients with a higher dosage of 30%, while anthracyclines were reduced in 20% of cases with a higher dosage of 30%. As these reductions occurred in the first three months of treatment, they could probably be linked to suspected adverse reactions that are regarded as ADRs.

An interesting issue that could be evaluated in another study is whether PFS and OS was affected in patients who underwent a dose reduction of more than 30%.

Our study highlights a very interesting phenomenon regarding the difference between the interruption of 5-FU and capecitabine. We observed that patients treated with capecitabine interrupted treatment much more frequently than those receiving 5-FU. In fact, capecitabine was discontinued in 17.1% of cases compared to only 6.3% of patients receiving 5-FU. In fact, several studies comparing capecitabine + oxaliplatin and 5-FU + oxaliplatin concluded that the two treatments are comparable in terms of both efficacy and toxicity [9–13]. Such findings highlight the difference between published data and our post-marketing data, which confirmed that capecitabine was interrupted more frequently because of severer toxicity.

Ifosfamide was the most frequently reduced drug, one in three patients stopping chemotherapy due to an adverse event. A decidedly different issue (problem) is that of nephrotoxicity during treatment. It has been reported to be responsible for clinical nephrotoxicity in around 30% of patients, although about 90% of cases also manifest subclinical tubular toxicity with glycosuria and β2-microglobulinuria [14]. In our study, the interruption of ifosfamide was due to the development of renal failure.

In routine clinical practice, dose reduction or treatment interruption is sometimes necessary when the risk-benefit ratio of the drug is not worth for the patient. Such action may also be taken in the event of unexpected side-effects or complications/reactions that have not as yet been identified as a side-effect of the drug in question. This may happen because phase I, II and III studies generally analyze no more than 2000 patients. This only allows us to recognize common side effects, while uncommon side effects are often not recognized in the early stages of experimentation. Uncommon side-effects are those reported in less than 1 case per thousand population and it is the task of pharmacovigilance to identify them. Although rare, the clinician must nevertheless be aware of these events. In addition, patients enrolled onto clinical trials are selected and do not reflect the patient population. Generally, in the studies, patients are more selected. Elderly patients and patients affected by heart disease are excluded from studies. This patients are, however, normally present in clinical practice. Therefore pharmacovigilance system is very important for report any toxicities in all categories of patients.

The main limitation of our study was the lack of clinical data available, which led to difficulties in analyzing the types of patients involved in the study. It was not therefore possible to identify the clinical features that may have contributed to the onset of the adverse reactions studied.

In conclusion, this study shows that post-marketing drug studies should be conducted in a more in-depth, systematic way, thus allowing us to evaluate both the effectiveness and the tolerance levels of the treatments. A program of active pharmacovigilance such as the one we have created at our institute is essential if the objective is to continuously monitor cancer drugs. In our study, by identifying patients who discontinued treatment we were able to discern adverse reactions which were then included in the National Network of Pharmacovigilance. A program of prospective, active pharmacovigilance will provide us with the opportunity of identifying and reporting more significant adverse events.

## MATERIALS AND METHODS

This retrospective observational study was designed to monitor critical phenomena, including dose reduction and treatment interruption, as potential indicators of ADRs. The following centers were involved in the study: IRST IRCCS, Meldola, Ravenna, Rimini and Reggio Emilia Hospitals, and the University Hospitals of Bologna, Ferrara and Parma. All consecutive cancer patients treated with non-experimental cancer drugs in adjuvant, locally advanced and metastatic settings were included in the study. Dose reductions were taken into consideration from 1^st^ January 2012- 31^st^ March 2013. Treatment discontinuation was evaluated from 1^st^ January 2012 to 28^st^ February 2012 as this was considered long enough to highlight any interruptions caused by adverse reactions. Exclusion criteria were as follows: treatment regimens lasting ≤ 60 day; treatment discontinuation because of planned radiotherapy; non attendance for treatment; and treatment discontinuation due to surgery.

Intravenous (5-fluorouracil, oxaliplatin, carboplatin, docetaxel, paclitaxel, rituximab, pemetrexed, doxorubicin, ifosfamide) and oral (erlotinib, sorafenib, lenalidomide, capecitabine) drugs were considered for the study.

All data were retrieved from the institute's prescription pharmacy database (Log80).

Although no formal statistical hypotheses were formulated due to the explorative nature of this study, appropriate descriptive statistics were performed.

We analyzed the frequency of >10% dose reduction during the first 90 days of treatment, calculated on the basis of the actual dose administered to the patient rather than the dose prescribed according to the regimen. Ninety days were calculated as an appropriate length of time in which an adverse reaction might occur. Treatment interruption was calculated on the basis of the disappearance of the patient's name from the pharmacy database within 60 days of the first prescription.

We used a regional administrative database. The study database was anonymized by deleting the identity of the patients and other sensitive information and by assigning a unique numerical code to each individual. When anonymized administrative data are used for healthcare planning, studies are exempt from formal ethics review and specific written consent is not required to use patient information stored in hospital databases.

All the authors of this manuscript are affiliated with the centers that participated in the multicentric study. They received the anonymous data from electronic archives. None of the authors had direct contact with patients at any time during the study.

The project was funded by AIFA (Italian Medicines Agency, Regional Note PG / 2011/194626 of 08.08.2011).
